# The Role of External Inputs and Internal Cycling in Shaping the Global Ocean Cobalt Distribution: Insights From the First Cobalt Biogeochemical Model

**DOI:** 10.1002/2017GB005830

**Published:** 2018-04-16

**Authors:** Alessandro Tagliabue, Nicholas J. Hawco, Randelle M. Bundy, William M. Landing, Angela Milne, Peter L. Morton, Mak A. Saito

**Affiliations:** ^1^ School of Environmental Sciences University of Liverpool Liverpool UK; ^2^ MIT‐WHOI Joint Program in Oceanography/Applied Ocean Science and Engineering, Department of Marine Chemistry and Geochemistry Woods Hole Oceanographic Institution Woods Hole MA USA; ^3^ Department of Earth Sciences University of Southern California Los Angeles CA USA; ^4^ Marine Chemistry and Geochemistry Woods Hole Oceanographic Institution Woods Hole MA USA; ^5^ School of Oceanography University of Washington Seattle WA USA; ^6^ Department of Earth, Ocean, and Atmospheric Science Florida State University Tallahassee FL USA; ^7^ School of Geography, Earth and Environmental Sciences University of Plymouth Plymouth UK; ^8^ Geochemistry, National High Magnetic Field Laboratory Tallahassee FL USA

**Keywords:** biogeochemistry, trace elements, modeling

## Abstract

Cobalt is an important micronutrient for ocean microbes as it is present in vitamin B_12_ and is a co‐factor in various metalloenzymes that catalyze cellular processes. Moreover, when seawater availability of cobalt is compared to biological demands, cobalt emerges as being depleted in seawater, pointing to a potentially important limiting role. To properly account for the potential biological role for cobalt, there is therefore a need to understand the processes driving the biogeochemical cycling of cobalt and, in particular, the balance between external inputs and internal cycling. To do so, we developed the first cobalt model within a state‐of‐the‐art three‐dimensional global ocean biogeochemical model. Overall, our model does a good job in reproducing measurements with a correlation coefficient of >0.7 in the surface and >0.5 at depth. We find that continental margins are the dominant source of cobalt, with a crucial role played by supply under low bottom‐water oxygen conditions. The basin‐scale distribution of cobalt supplied from margins is facilitated by the activity of manganese‐oxidizing bacteria being suppressed under low oxygen and low temperatures, which extends the residence time of cobalt. Overall, we find a residence time of 7 and 250 years in the upper 250 m and global ocean, respectively. Importantly, we find that the dominant internal resupply process switches from regeneration and recycling of particulate cobalt to dissolution of scavenged cobalt between the upper ocean and the ocean interior. Our model highlights key regions of the ocean where biological activity may be most sensitive to cobalt availability.

## Key Points

- The first global model of ocean cobalt cycling is presented
- Sediments are the major global source of cobalt
- Suppression of Co scavenging by low oxygen and reduced bacterial activity is key in extending the residence time of externally supplied Co

## 1 Introduction

When compared to typical phytoplankton requirements, cobalt (Co) emerges as being relatively depleted in seawater (Moore et al., 2013; Saito et al., 2008), and in some ocean regions, there is evidence that Co is the primary or secondary limiting nutrient (Bertrand et al., 2007, 2015; Browning et al., 2017; Sañudo‐Wilhelmy et al., 2006). Marine phytoplankton requires Co due to its presence in vitamin B_12_ (also known as cobalamin) and due to its role as a potential cofactor of carbonic anhydrase and alkaline phosphatase, which catalyze carbon fixation and organic phosphorus acquisition, respectively (although a confirmation awaits of a marine pelagic microbe with Co within its alkaline phosphatase; Wojciechowski et al., 2002). In eukaryotic algae, zinc (Zn) can be substituted for Co in carbonic anhydrase and alkaline phosphatase when Co levels are low (Morel et al., 1994; Sunda & Huntsman, 1995), but cyanobacteria are known to have an obligate Co requirement (Saito et al., 2002). Despite the Co‐containing vitamin B_12_ playing a fundamental role in the development of the first theories for algal nutrient limitation and its representation in numerical models (Droop, 1973, 1974), Co cycling remains ignored in contemporary global ocean biogeochemical models.

While early pioneering studies considered Co to be a “scavenged‐type” element (Jickells & Burton, 1988), subsequent investigations with lower detection limits identified the nutrient‐like depletion in the upper photic zone highlighting the role of biological Co uptake (Martin et al., 1989; Noble et al., 2008; Saito & Moffett, 2002). Thus, Co is better described as a “hybrid‐type” element, with external inputs from continental margins (in particular within the major oxygen minimum zones), as well as riverine, and dust sources (Noble et al., 2012; Saito & Moffett, 2002; Saito et al., 2004; Shelley et al., 2012; Zhang et al., 1990). In addition to these external inputs, Co is strongly removed from the dissolved fraction via scavenging (Bruland et al., 2014; Moffett & Ho, 1996). The scavenging of Co is catalyzed by manganese (Mn) oxidizing bacteria, due to the similar ionic radii and redox potentials of Mn and Co (Cowen & Bruland, 1985; Moffett & Ho, 1996; Sunda & Huntsman, 1988).

Like many bioactive metals, Co is often found strongly bound by natural ligands, particularly in the upper water column (Ellwood & van den Berg, 2001; Saito & Moffett, 2001). These ligands have been shown to be produced from cyanobacteria blooms and released to the ocean upon cell lysis (Saito et al., 2005) and are hypothesized to be important in protecting Co from scavenging and in decreasing Co bioavailability to some phytoplankton when complexed (Moffett & Ho, 1996; Saito et al., 2002). Yet unlike other bioactive metals (e.g., iron), in some regions, Co can also be found to be unsaturated by natural ligands, particularly in coastal and polar regions. This results in measurable concentrations of labile Co that are likely far more bioavailable (Saito et al., 2004, 2010). Thus far, the only known Co ligands are likely to be the cobalamins that are known to be synthesized by some bacteria (including cyanobacteria) and archaea.

With the arrival of GEOTRACES research cruises at the ocean basin scale, a number of studies have provided detailed data on the distributions of Co in the Atlantic, Pacific, and Southern Oceans. The zonal sections identified major plumes of Co in the major oxygen‐depleted regions of the North and South Atlantic and South Pacific Oceans, extending well into each basin and farther than analogous iron or Mn plumes (Hawco et al., 2016; Noble et al., 2012, 2017), implying lesser Co scavenging. In addition, many of these studies have observed widespread depletions of dissolved (dCo) in the upper ocean, typical of its role as a micronutrient, even in the dust‐laden North Atlantic Ocean (Bown et al., 2011; Dulaquais et al., 2014; Hawco et al., 2016; Noble et al., 2012, 2017). These large‐scale oceanographic Co data sets have facilitated the examination of the links between Co and other parameters, such as positive links with phosphate and nitrous oxide within the euphotic zone and inverse relationships with dissolved oxygen in the mesopelagic (Noble et al., 2012, 2017). When compared to phosphate, Co is distinguished from other bioactive metals (e.g., cadmium or zinc) in having a wide range of Co:P slopes that span more than an order of magnitude (Bown et al., 2011; Noble et al., 2017; Saito et al., 2010; Saito & Moffett, 2002), suggestive of variable microbial use. In certain ocean regions, Co section data sets spanned regions known to contain hydrothermal plumes of iron and Mn (Hatta et al., 2015; Resing et al., 2015; Saito et al., 2013), but no corresponding Co plumes were observed (Hawco et al., 2016; Noble et al., 2012, 2017).

At the global scale, ocean biogeochemical models are excellent platforms with which to explore the differing roles of often competing signals linked to external inputs and internal cycling in different biogeochemical regimes and water masses. In this study, we developed the first representation of Co cycling in a global ocean model and investigated how external inputs and internal cycling shape the oceanic distribution of this important micronutrient. We find key roles played by low oxygen and the suppression of bacterial activity by temperature in promoting the longevity of Co. This then facilitates the widespread impact of Co inputs from the ocean margins at the basin scale.

## 2 Methods

We have devised the first global Co model that is coupled to the PISCES‐v2 model, which is itself coupled to offline circulation fields within the NEMO framework (http://www.nemo-ocean.eu). The PISCES‐v2 model simulates a wide range of tracers: nitrate, ammonium, phosphate, silicic acid, iron, iron‐binding ligands, dissolved oxygen, two size classes of particles, two phytoplankton functional types (diatoms and nanophytoplankton), two grazers, dissolved organic carbon, dissolved inorganic carbon, biogenic silica, calcium carbonate, and alkalinity (Aumont et al., 2015; Tagliabue & Resing, 2016). In this work, we have augmented PISCES‐v2 with an additional six tracers to resolve the biogeochemical cycling of Co. The additional tracers are dissolved cobalt (dCo), scavenged cobalt (scCo; putatively associated with Mn oxides), cobalt within diatoms (PhyCo_D_), cobalt within nanophytoplankton (PhyCo_N_), small particulate organic cobalt (PCo_S_), and large particulate organic cobalt (PCo_B_). The Co within microzooplankton and mesozooplankton is an inferred quantity driven by a fixed Co/P ratio within zooplankton, which then drives excretion of Co when prey Co is greater than the required Co, as for Fe in PISCES‐v2 (Aumont et al., 2015). All parameter values for the Co model are described in Table 1.

**Table 1:** Model Parameter Values

Parameter	Value	Units	Description
O_2thres_1_	50	μM O_2_	Threshold for enhanced sedimentary Co fluxes
O_2thres_2_	2	μM O_2_	Threshold for eliminated sedimentary Co fluxes
*θ*_MAX_	150/150	μmol Co/mol P	Maximum phytoplankton Co quotas
kdCo	50/80	pM Co	Half saturation constants for Co uptake
KZnCo	0.5	nM Zn	Half saturation constant for Zn‐Co interaction for diatom group only
ΛCo_MIN_	0.1 × 10^−3^	day^−1^	Minimum dCo scavenging rate
ΛCo	0.01	day^−1^	Maximum dCo scavenging rate
O_2ST_	100	μM O_2_	Threshold for O_2_ effect on scavenging
kO_2_Λ	25	μM O_2_	Half saturation constant for O_2_ effect on scavenging and dissolution
kBΛ	2.5	μM C	Half saturation constant for bacterial effect on scavenging
kPARΛ	15	W/m^2^	Half saturation constant for PAR effect on scavenging
O_2DT_	50	μM O_2_	Threshold for O_2_ effect on sCo dissolution
*λ*_MAX_	0.1	day^−1^	Maximum sCo dissolution rate
CoL_MIN_	25	pM	Minimum concentration of Co ligands
*θ*_ZOO_	20	μmol Co/mol P	Zooplankton Co quota
*ϕ*	1.5	Unitless	Relative rate of particulate organic Co remineralization

*Note*. Where two values are given, the first is for nanophytoplankton and the second is for diatoms.

### 2.1 Generalized Source‐Sink Equations for Co Tracers

(1) ddCodt=DustCo+SedCo+RiverCo−UpCo−ScavCo+DissolCo+ExcretCo+ReminCo

dCo is supplied from dust (Dust_Co_), sediments (Sed_Co_), and rivers (River_Co_), with no source from hydrothermal venting based on results from GEOTRACES sections. dCo is consumed by phytoplankton (Up_Co_) and lost due to scavenging (Scav_Co_). dCo is also resupplied from the dissolution of scCo (Dissol_Co_) and excretion by zooplankton (Excret_Co_) and the remineralization of particulate organic cobalt (Remin_Co_).

(2) dscCodt=ScavCo−DissolCo−sinking

scCo is produced due to the scavenging of dCo (Scav_Co_), and scCo dissolves via dissolution back to dCo (Dissol_Co_). scCo sinks at 1 m/day.

(3) dphyCoidt=UpCoi−SMSi

Phytoplankton Co of functional group *i* (D = diatom or N = nanophytoplankton) is a result of dCo uptake (Up_Co_), which is explicitly modeled and specific to diatoms and nanophytoplanktons (Up_CoD_ and Up_CoN_, respectively; Up_Co_ in equation (1) is the sum of both terms). Loss of phytoplankton Co follows the processes of mortality, aggregation, and grazing (SMS_*i*_) in the main PISCES‐v2 model (Aumont et al., 2015).

(4) dPCoidt=SMSi−ReminCo

Remineralization of Co from small and large organic Co particles (subscript *i* = S or B, Remin_CoS_ and Remin_CoB_, respectively; Remin_Co_ is the sum of both terms) is modeled independently via a tunable parameter relative to the remineralization of organic carbon (*ϕ*). By default, we assume particulate organic Co remineralizes 50% faster than organic carbon (*ϕ* = 1.5). Other gains and loss of particulate Co (SMS_S_ and SMS_B_) follow the processes of aggregation, disaggregation, phytoplankton and zooplankton mortality, sinking, and grazing as per the main PISCES model (Aumont et al., 2015).

### 2.2 External Inputs of Co

Dust input assumes a mineral fraction of Co of 17.3 μg/g (Rudnick & Gao, 2014) and a Co solubility of 8% (Shelley et al., 2012). River supply assumes a Co/C ratio of 12 μmol/mol (Gaillardet et al., 2003). Sediment Co input (sed_Co_) is modeled via a set of bottom water oxygen (O_2bw_) dependent thresholds and is benchmarked to the sedimentary iron (Fe) supply (sed_Fe_) in the model, which is a function of organic carbon flux to the sediment:

$$\begin{array}{l} O_{2\mathrm{bw}}\leq O_{2\text{thres}\_1},{\mathrm{sed}}_{\mathrm{Co}}={\mathrm{sed}}_{\mathrm{Fe}}\times M\times 1,000\\ O_{2\mathrm{bw}}\leq O_{2\text{thres}\_2},{\mathrm{sed}}_{\mathrm{Co}}=0\\ O_{2\mathrm{bw}}>O_{2\text{thres}\_1},{\mathrm{sed}}_{\mathrm{Co}}={\mathrm{sed}}_{\mathrm{Fe}}\times M\times 25 \end{array}$$

where *M* represents the Co/Fe mineral fraction ratio (Rudnick & Gao, 2014). The 1,000 and 25 factors are tuned to account for suboxic Co release from dissolution of Fe and Mn oxides under suboxic conditions and Co incorporation into Fe sulfides (pyrite) under very low O_2_ when sulfate reduction initiates in porewaters of surface sediments. Specifically, O_2thres_1_ = 50 μM O_2_ and O_2thres_2_ = 2 μM O_2_.

In oxic sediments, Co in porewaters is very low with <2.5 nM (Heggie and Lewis, 1984), due to rapid Mn oxidation in near‐surface sediments where O_2_ from the water column can penetrate. Oxidation of Co in microbially catalyzed Mn oxidation (Lee & Fisher, 1993; Moffett & Ho, 1996) limits the diffusive flux out of sediments substantially. Co/Al ratios in continental margin sediments (e.g., South China Sea; Hu et al., 2012; 2013) reflects crustal Co/Al ratios, likely because most of the Co released by dissolution/weathering/desorption of crustal materials is returned to the sediments via Mn oxidation in estuaries and coastal seas (i.e., is scavenged; Hawco et al., 2016; Moffett & Ho, 1996).

The small flux when O_2_ > O_2_thres_1_ reflects release by sediment desorption and/or ligand stabilization of Co in estuaries (Bewers & Yeats, 1977; Kharkar et al., 1968; Zhang et al., 1990). Suboxic release of Co has been shown explicitly (Johnson et al., 1988; Sundby et al., 1986) and is reflected in low Co/Al ratios in margin sediments in OMZs off Peru (Böning et al., 2004), Chile (Böning et al., 2009), the Gulf of California (Brumsack, 1989), and in the South Atlantic under the Benguela upwelling region (Bremner & Willis, 1993). Co fixation into pyrite formation follows thermodynamic predictions (e.g., Morse & Luther, 1999; Saito et al., 2003) and can be seen from high Co/Al ratios in Black Sea sediments (Brumsack, 2006) and in sulfide‐rich sediments near Walvis Bay on the Namibian Coast (Borchers et al., 2005). The precise choice of the threshold concentrations chosen here reflects model tuning to the dissolved oxygen concentrations in the model, balancing model performance in the Atlantic, Pacific, and Indian Oceans.

### 2.3 Internal Cycling of Co

#### 2.3.1 Phytoplankton Uptake

Phytoplankton uptake of Co is explicitly modeled rather than using a “Redfield” conversion based on the modeled carbon fixation rate. Equation (5) represents this in a similar manner to how PISCES models Fe uptake, accounting for a maximum cellular quota and the potential for uptake to be upregulated under certain conditions (Aumont et al., 2015). This decouples Co uptake from C fixation and permits variable Co/C ratios as observed (Sunda & Huntsman, 1995).

(5) $${\mathrm{Up}}_{\mathrm{Coi}}=\mu_{\mathrm{MAX}i}\theta_{\mathrm{MAX}i}\mathrm{bCo}/\left(\mathrm{bCo}+\text{kbCoi}\right)\frac{\left(1-\mathrm{\theta i}/,\theta_{\mathrm{MAX}i}\right)}{\left(1.05-\mathrm{\theta i}/,\theta_{\mathrm{MAX}i}\right)}\xi \mathrm{Zn}$$

where subscript *i* denotes either D (diatoms) or N (nanophytoplankton), bCo is the bioavailable Co pool and is assumed to represent dCo for nanophytoplankton (based on observations/assumptions that cyanobacteria utilize both CoL complexes and Co′; Saito et al., 2002) and inorganic cobalt species, Co′, for diatoms (based on observations by Sunda & Huntsman, 1995; see below for the calculation of Co′), and *μ*_MAX*i*_ is the maximum growth rate of functional type *i*. The Co/P ratio is represented by *θ*_*i*_ within the functional group *i*, and *θ*_MAX*i*_ is the maximum Co/P ratio for phytoplankton functional group *i*, while kbCo*i* is the half saturation constant for bCo uptake for functional group *i*. Co uptake is downregulated when *θ* approaches *θ*_MAX_ using a hyperbolic function with a shape factor set to 0.5 (as for Fe in PISCES‐v2). The term *ξ*_Zn_ is a scalar active only for diatoms and represents the interreplacement of Co and Zn within carbonic anhydrase causing Zn concentrations to affect Co uptake (Price & Morel, 1990; Saito & Goepfert, 2008; Sunda & Huntsman, 1995; Xu et al., 2007) via

(6) $$\xi_{\mathrm{Zn}}=\mathrm{MAX}\left[0.1,\;3\times \left(1-\frac{\mathrm{Zn}}{\left(\mathrm{Zn}+\text{kZnCo}\right)}\;\right)\right]$$

where kZnCo represents the half saturation constant for Zn‐Co interactions. This is initially set to 0.5 nM Zn, which would approximately reflect a free Zn concentration of 5 pM. Previous work has shown enhanced Co uptake when Zn falls below 5–10 pM in several species of eukaryotic phytoplankton (Sunda & Huntsman, 1995). In the absence of a specific Zn model, the Zn concentration (in nM) is derived from Si (in μM) using 0.065 × Si + 0.183 (M. C. Lohan, personal communication, 2017) as there is a long noted relationship between Zn and Si (Bruland et al., 1978). The constants 0.1 and 1.3 in equation (6) decrease Co uptake by up to 90% when Zn is abundant and increase Co uptake up to threefold when Zn is scarce, respectively.

#### 2.3.2 Scavenging and Dissolution

The scavenging of dCo is assumed to be driven by Mn oxides produced by Mn‐oxidizing bacteria (Johnson et al., 1988; Moffett & Ho, 1996). At this stage, our model does not include an explicit Mn module, so we assume Mn oxides to be prevalent where oxygen is abundant (Ohnemus et al., 2017; Ohnemus & Lam, 2015) and that the activity of heterotrophic Mn‐oxidizing bacteria scales with total bacterial activity (Cowen & Bruland, 1985; Moffett & Ho, 1996; Sunda & Huntsman, 1988), except in the surface ocean where manganese oxides are destroyed via photoreduction and dissolution (Sunda & Huntsman, 1988). Co loss is generally controlled by biological uptake in oligotrophic regions (Moffett & Ho, 1996). Although the factors controlling Mn oxidation remain poorly understood (Lee & Fisher, 1993), Mn oxidation is the likely vector for Co scavenging given (1) the known ability for Co to be co‐oxidized by Mn‐oxidizing bacteria (Lee & Fisher, 1993; Moffett & Ho, 1996); (2) similar redox potentials and ionic radii of Co and Mn (Moffett & Ho, 1996; Swanner et al., 2014); (3) extensive covariation between Co and Mn contents of solid‐phase marine sediments, manganese nodules, and ferromanganese crusts (Krishnaswami, 1976; Manheim, 1986), which accumulate Co scavenged from the water column; and (4) covariation of particulate Co and Mn phases in the mesopelagic (Saito et al., 2016). In addition to oxygen‐related cycling of Mn‐oxides in sediments, the absence of particulate Mn has been long noted in offshore oxygen minimum zones of the North and South Pacific (Johnson et al., 1996; Landing & Bruland, 1987; Ohnemus et al., 2017) and attributed to slow Mn oxide formation at low O_2_ and in situ reduction.The specific rate of scavenging (Λ) is based on a minimum (ΛCo_min_) and maximum scavenging rate (ΛCo) that is modulated by oxygen, bacterial activity (itself affected by nutrient and dissolved organic matter limitation), and light:

(7) $$\Lambda =\Lambda_{\mathrm{MIN}}+\mathrm{\Lambda Co}\times Q\times kO_{2}\times \text{kBACT}\times \left(1-\text{kPAR}\right)\;$$

*Q* is the specific temperature function for Co oxidation by manganese‐oxidizing bacteria with a *Q*_10_ of 2.75 (Lee & Fisher, 1993). The various other terms relate to the impact of oxygen, bacterial activity, and light on Co scavenging:

(8) kO2=O2−O2ST2O2−O2ST2+kO2Λ2

where O_2_ is dissolved oxygen, O_2ST_ is the threshold concentration for scavenging, and kO_2_Λ is the half saturation constant for the influence of O_2_ on Co scavenging.

(9) kBACT=BACT2BACT2+kBΛ2

BACT is the biomass of bacteria (μM C; see Aumont et al., 2015) and kBΛ is the half saturation constant for the influence of bacterial activity on Co scavenging;

(10) kPAR=PAR2PAR2+kPARΛ2

where PAR is photosynthetically active radiation and kPARΛ is the half saturation constant for the influence of irradiance on Co scavenging. The overall loss of dCo (Scav_Co_) is then governed by the scavenging rate (Λ) and the Co prime concentration (Co′) such that Scav_Co_ = Λ × Co′.

Since Cobinding ligands are very strong, with log*K*_cond_ > 16 (Ellwood & van den Berg, 2001; Saito & Moffett, 2001; Saito et al., 2005), and are found at concentrations that are less than or equal to the dissolved Co concentration, we determine the Co prime (Co′) concentration via Co′ = dCo − CoL, where Co′ is defined as the sum of inorganic cobalt complexes and Co^2+^. It should be noted that if there are any weaker organic Co complexes below the detection window of voltammetric studies, then they are part of the labile Co reservoir, which is a measured Co parameter that is often compared with Co′. The oxidation state of Co is not explicitly calculated within the model; however, empirical detection window studies have found that CoL complexes must be in the Co(III) state and solubility estimates require that Co′ is Co(II) (Saito et al., 2005).

Co ligands have been observed to be produced by communities dominated by picocyanobacteria (Saito et al., 2005) and could be sourced from release of intracellular cobalamin/pseudocobalamin cofactors, or their precursors and photodegradation products, during the grazing or viral lysis of microbes in euphotic zone as part of the microbial loop. All sequenced marine cyanobacteria have the genes required for the de novo biosynthesis of pseudocobalamin, starting from inorganic Co species (Helliwell et al., 2016). We therefore link the production of Co ligands to the relative abundance and biomass of nanophytoplankton in our model, although future efforts could consider additional prokaryotic sources. Co ligands have an imposed minimum concentration (CoL_MIN_) to stabilize dCo in the deep ocean. At present, this component of the model is a simple means to represent Co speciation and does not permit any excess Co binding ligands, although their presence remains debated (Ellwood et al., 2005; Saito & Moffett, 2001; Saito et al., 2005). The loss of dCo via scavenging is then Co′ × Λ. At Co′ concentrations greater than 100 pM, Co′ is lost at an elevated rate (10 × ΛCo). Dissolution of scCo occurs where light is high or O_2_ is low:

(11) $$\lambda =\lambda_{\mathrm{MAX}}\times \mathrm{MAX}\left[\text{kPAR},kO_{2}d\right]$$

where kPAR is as per equation (10) and

(12) kO2d=O2−O2DT2O2−O2DT2+kO2Λ2

where O_2DT_ is the threshold concentration for dissolution and *λ*_MAX_ is the specific rate of scCo dissolution. The specific rate of scCo dissolution is then multiplied by scCo to result in Dissol_Co_.

#### 2.3.3 Excretion and Remineralization

Zooplankton excretion of Co is modeled in the same manner as for Fe in PISCES‐v2 and is accordingly enhanced when prey are rich in Co, relative to the imposed zooplankton Co quota (Table 1). The remineralization of organic Co, relative to organic P, can be upregualted or downregulated by the scalar *ϕ*. During model tuning, a 50% faster remineralization rate for Co, relative to P, was found to improve the Atlantic‐Pacific contrast.

### 2.4 Model Experiments and Data Sets

The standard Co model (CTL) was spun up for 1,000 years, and a range of different experiments were then conducted for 125 years each. To determine the role of specific source processes, we ran experiments with no dust supply (NODUST) and no sediment supply (NOSED). In addition, the effect of oxygen thresholds on coastal sources and scavenging was investigated. We ran experiments where low oxygen did not enhance sediment Co fluxes (NOSEDOX; i.e., where O_2thres_1_ = 0) and where low oxygen did not switch off sedimentary Co fluxes (NOSEDOXA; i.e., where O_2thres_2_ = 0). We then ran a set of experiments where oxygen did not affect scavenging rates (SCAV1) and where bacterial activity did not affect scavenging rates (SCAV2).

To assess our Co model, we compiled 8,235 Co data points from a variety of studies. They are compared graphically with the model results as raw data. For the statistical comparisons, the Co observations are gridded on to a 1° × 1° horizontal grid with 33 vertical levels (bounded by 0, 10, 20, 30, 40, 50, 75, 100, 125, 150, 200, 250, 300, 400, 500, 600, 700, 800, 900, 1,000, 1,100, 1,200, 1,300, 1,400, 1,500, 1,750, 2,000, 2,500, 3,000, 3,500, 4,000, 4,500, 5,000, and 5,500 m) to compare directly with the model results on the same grid. We pay particular attention to examining the distributions of dCo along key GEOTRACES and CLIVAR transects in the Atlantic, Pacific, and Indian Oceans to evaluate model performance.

Data from the GEOTRACES GA03, CoFeMUG (GAc01), and GP16 sections are measured by cathodic stripping voltammetry following UV oxidation to destroy organic Co ligands. An analytical ligand, dimethyl glyoxime, binds to cobalt and is reduced with Co at a defined potential of −1.15 V, resulting in a reduction peak proportional to the Co concentration. Sample‐specific instrument sensitivity is determined with four successive 25‐pM standard additions, and a blank is subtracted. SAFe and GEOTRACES community standards are measured to ensure comparability with other methods.

Dissolved Co concentrations in samples from CLIVAR lines I8/I9, P16, and I5 were measured using flow‐through solid phase extraction systems at FSU/NHMFL (Milne et al., 2010). For all cruises, samples were UV oxidized for 1.5 hr to destroy organic Co ligands and permit total extraction of Co by the chelating resin (Toyopearl AF‐Chelate‐650M for P16, Nobias Chelate PA‐1 for I8/I9 and I5). Sample aliquots (10–20 ml, 0.024 M HCl) were buffered to pH ~6 with ammonium acetate and flowed through a resin column at 2 ml/min. The captured Co was eluted from the resin using 0.5–1 ml of 1.0 M HNO_3_ (UHP) and analyzed using high‐resolution inductively coupled mass spectrometry (Thermo ELEMENT 2, NHMFL/FSU). Concentrations were quantified using standard additions, blank‐corrected using aliquots of similarly extracted UHP water (18.2 MΩ cm), and verified through SAFe (S1 and D2) and GEOTRACES (GS and GD) community standards.

## 3 Results and Discussion

### 3.1 Modeled Co Distribution

**Figure 1:**
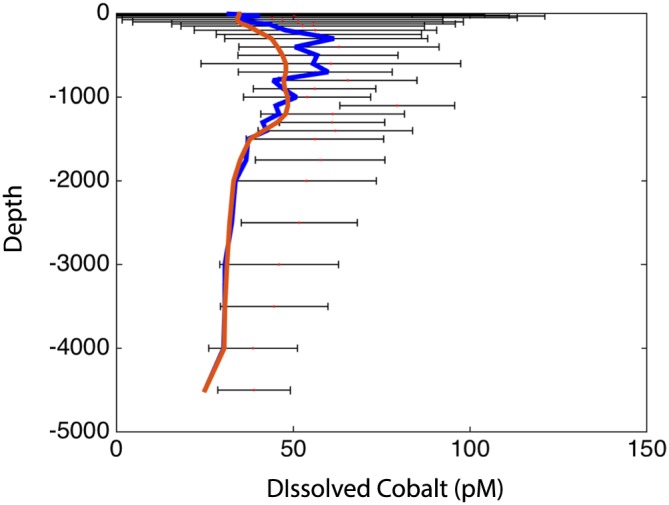
Global cobalt data vertical distribution. Bars represent the standard deviation around the mean cobalt (pM) from each vertical bin (see methods). The blue line represents the model mean vertical profile extracted at the same location as the data while the red line represents the overall mean vertical profile of the model.

We have compiled 8,235 dCo observations from the major ocean basins to serve as a basis for evaluating the skill of the base model. In general, the data displays the known “hybrid” character of dCo in the ocean (Figure 1). The surface ocean dCo levels are low on average but show high variability, while in the ocean interior dCo increases from the surface to intermediate depth but then declines from the intermediate to deep ocean (Figure 1). The mean model dCo profile extracted at the same location as the observations does a good job in reproducing the observed behavior, exhibiting a parallel decline in mean dCo toward the surface ocean and the ocean interior from intermediate water depth (~1,000 m), with a broadly similar pattern shown by the overall modeled mean dCo (Figure 1).

**Figure 2:**
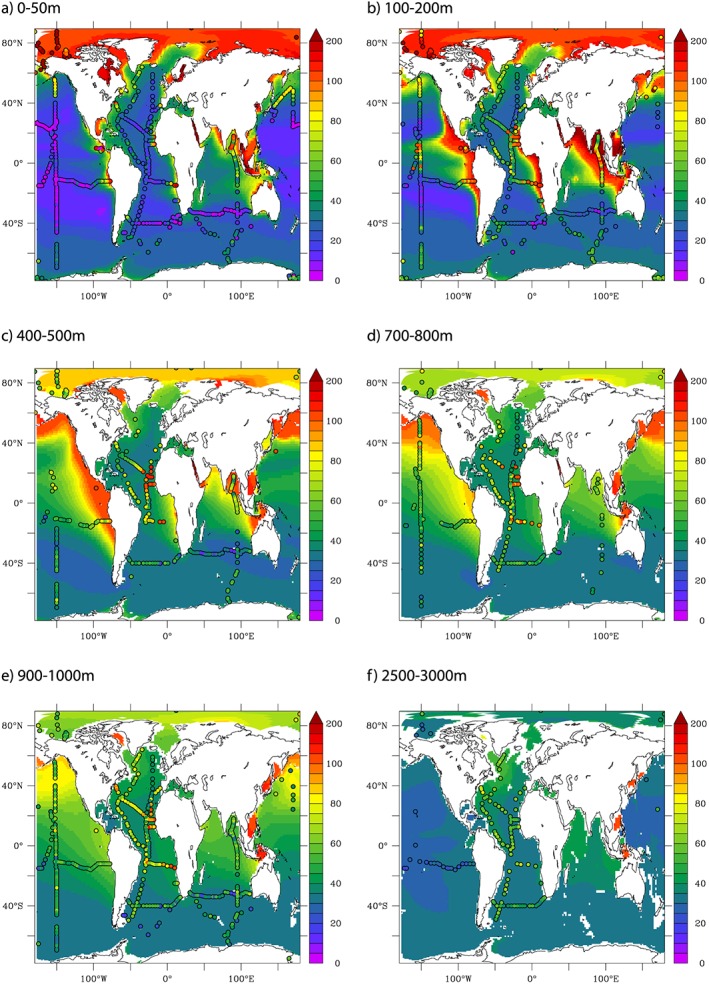
Observed versus modeled dCo (annual mean, pM) for different depth slices.

Spatially, the model is able to capture the major trends in dCo between different ocean regions and as a function of depth (Figure 2). As seen in observations, the Arctic Ocean is particularly dCo rich and declines with depth, whereas elsewhere dCo accumulates with depth, particularly so in the low oxygen regions of the tropical Atlantic, Pacific, and Indian Oceans. The major mismatch between the model and observations emerges in the Atlantic Ocean between 700–800 and 900–1,000 m, where the model underestimates the dCo levels within the low oxygen regions of the Mauritanian and Benguela upwelling areas. The CTL model has a correlation coefficient of >0.7 in the upper 200 m and >0.5 at depths greater than 900 m. The Atlantic mismatch between 700 and 800 m drives a correlation coefficient of 0.264 in this depth stratum (Table 3). Overall, the model has a correlation coefficient of 0.593 over the entire data set, which is comparable to the most skillful of global Fe models (Tagliabue et al., 2016).

**Figure 3:**
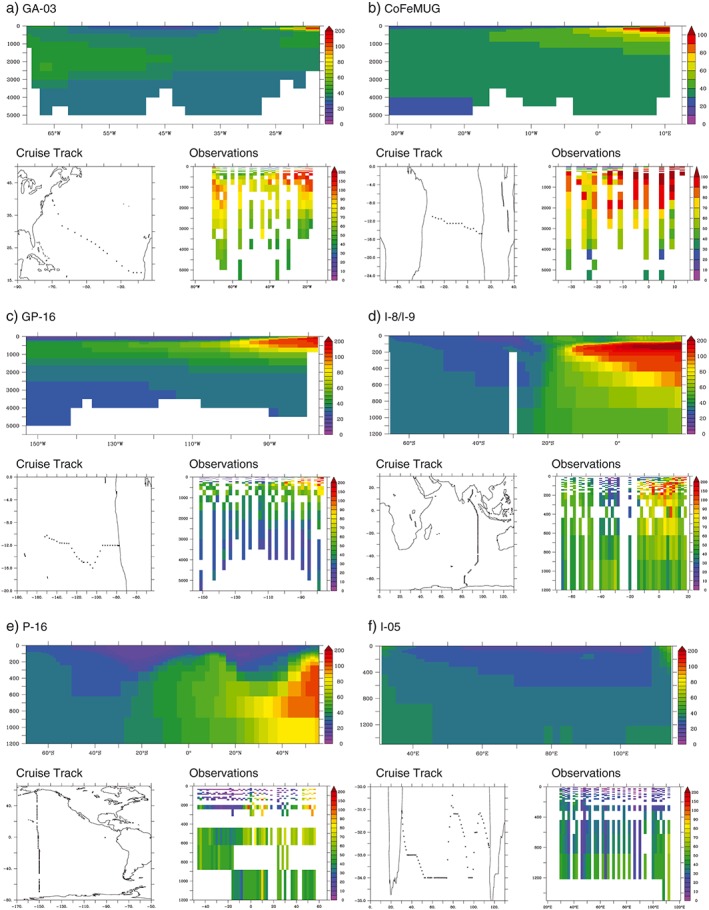
Observed versus modeled dCo (annual mean, pM) for specific GEOTRACES and CLIVAR sections.

Examining three GEOTRACES and three CLIVAR ocean sections reveals the strengths and weaknesses of the CTL model. On the GA‐03 transect between Woods Hole and Cape Verde in the North Atlantic subtropical gyre (Noble et al., 2017), the model is able to represent a dCo maxima associated with the eastern and western margins, but these are too muted relative to the data (Figure 3a). This is likely driven by dCo removal rates that are too high, too little dCo supply from ocean margins, or an underestimation of regeneration of Co from sinking organic matter (Noble et al., 2017). A similar result is found for the CoFeMUG section across the south subtropical Atlantic (Noble et al., 2012) where the margin enhanced dCo is produced by the model but remains less widespread than the data (Figure 3b). In the Pacific Ocean, the model does an excellent job of reproducing the large dCo plume observed on the GP‐16 transect (Hawco et al., 2016) and the low dCo upper ocean values (Figure 3c). The strong dCo maxima observed in the northern part of the Indian Ocean is well reproduced by the model along the CLIVAR I8 and I9 section (Figure 3d). The CLIVAR P16 section provides a unique window into the meridional distribution of dCo throughout the entire Pacific Ocean, and our model does a good job in reflecting the low dCo values in the surface ocean and gradual accumulation from south to north (Figure 3e). Finally, the zonal CLIVAR I05 section along the boundary between the Indian and Southern Oceans highlights the low dCo concentrations emanating from the dCo poor Southern Ocean (Figure 3f).

**Figure 4:**
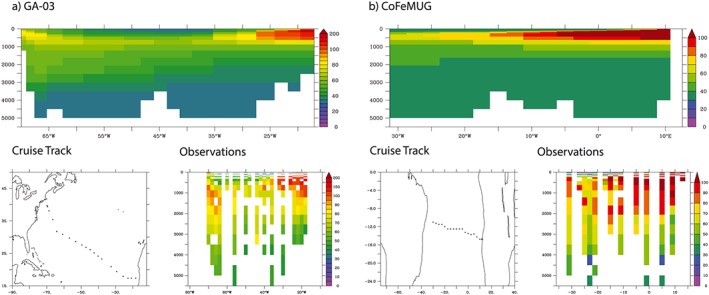
Observed versus modeled dCo (annual mean, pM) for two zonal Atlantic GEOTRACES sections when modeled oxygen is restored to World Ocean Atlas 2009 values.

The skill of the model in the Atlantic Ocean is related to the underlying biogeochemical model rather than the Co submodel. Initial tests aimed at examining whether Atlantic dCo could be enhanced by lowering scavenging rates (ΛCo) or enhancing sedimentary dCo fluxes led to unrealistic accumulations of dCo in the Pacific and Indian basins. These initial tests led us to examine whether the biogeochemical model was overestimating oxygen levels in the Atlantic low oxygen regions, which then led to elevated scavenging rates (via the oxygen dependence of equation (7)). This was quantified by running an additional experiment where model oxygen is annually restored to World Ocean Atlas climatological values. In this run we observe a marked improvement in the modeled dCo along the GA‐03 and CoFeMUG transects (Figure 4). Moreover, the model skill in the 700‐ to 800‐m depth stratum is enhanced twofold (Table 2). This further emphasizes the importance of oxygen in shaping dCo cycling in the ocean interior. There is relatively little change in the modeled dCo sections along the GP‐16 and CLIVAR I8 and I9 and P16 sections as the model already represents the low oxygen conditions well in these regions.

**Table 2:** Statistical Evaluation of the model.

Depth interval (m)	Standard model		World Ocean Atlas oxygen	
	*R*	RMSE	*R*	RMSE
0–50	0.791	4.6	0.803	5.7
100–200	0.735	5.0	0.800	5.0
400–500	0.480	10.7	0.712	6.3
700–800	0.264	23.6	0.578	7.0
900–1,000	0.530	18.2	0.442	11.7
2,500–3,000	0.607	13.2	0.478	11.3
				
0–5,500	0.593	8.7	0.596	6.9

*Note*. Correlation coefficient (*R*) and root‐mean‐square error (RMSE) for the standard model and a version of model where simulated oxygen is restored daily to World Ocean Atlas 2009 oxygen levels.

**Figure 5:**
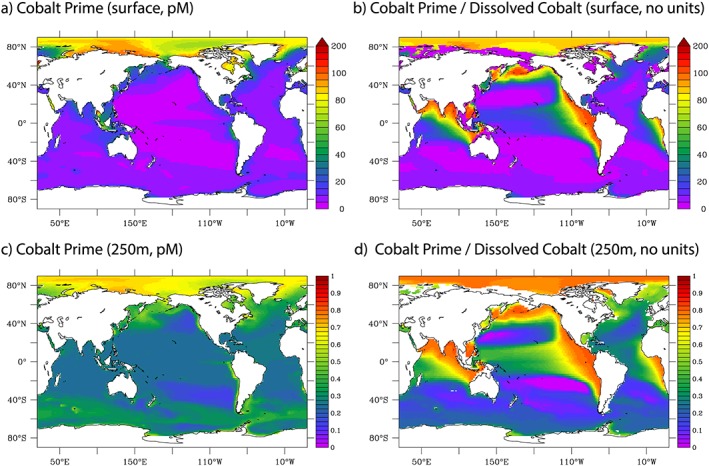
Free cobalt (pM) and the proportion of dCo that is free (no units) at the surface and at 250 m.

Our model also produces horizontal and vertical variations in the speciation of dCo. The greatest amounts of Co′ (defined as the sum of inorganic Co complexes species and Co^2+^) in both absolute and relative terms are found in the Arctic surface and interior waters and linked to the interior ocean oxygen minima of the tropical Atlantic, Pacific, and Indian oceans (Figure 5). These Co′ distributions are consistent with some high latitude observations (Saito et al., 2010) and is in some part controlled by Co organic complexes being linked to the prevalence of nanophytoplankton in the model (based on cyanobacterial evidence; Saito et al., 2002), which are less prevalent at high latitudes. Scavenging removal is the other component driving the accumulation of Co′. In our model, Co′ is removed by Mn‐oxidizing bacteria, and it is only where this process is impeded that Co′ can accumulate. In the oxygen minima, it is the low levels of oxygen that are restricting scavenging by Mn‐oxidizing bacteria, while in the Arctic Ocean and to some extent the Southern Ocean, it is instead the cold temperatures that lessen the scavenging of Co′ via lower bacterial metabolic rates. The ratio of Co′ to dCo in the model is often greater than 0.5 in the high dCo plumes in the ocean interior, which is a slight overestimate relative to the available data (Bown et al., 2012; Hawco et al., 2016; Noble et al., 2012).

### 3.2 Role of External Sources

**Figure 6:**
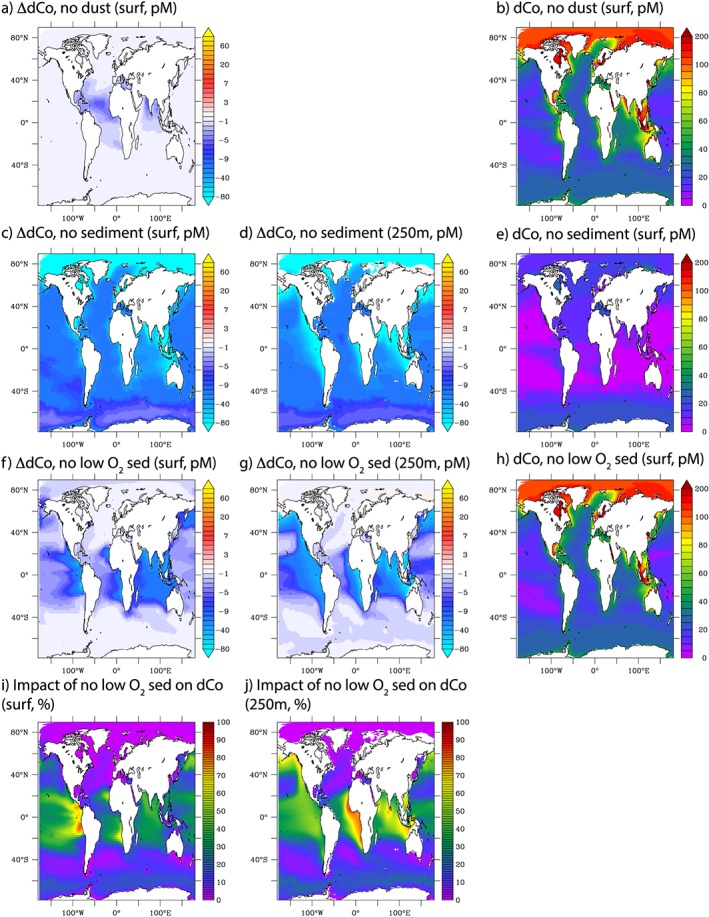
Absolute change in dCo (pM) at the surface (0–50 m) and at 250 m for no dust (a), no sediment supply (c and d), no sediment supply at low bottom water O_2_ (f and g), and the percentage of the total change due to sediment supply caused by low bottom water O_2_ sediment supply (i and j). (b, e, and h) The annually averaged surface dCo from the no dust, no sediment, and no sediment supply at low bottom water O_2_, respectively—please compare to Figure 2a.

Our sensitivity experiments permit an evaluation as to the role of different Co source processes in different geographic regions. Unsurprisingly, dust supply of Co is most important in the regions of the ocean typified by significant dust deposition from the Sahara, Namibian, and Arabian deserts. Nevertheless, the largest effects found for the tropical Atlantic rarely exceed 5 pmol/L in the upper 50 m (Figure 6a), and eliminating the dust Co source (NODUST) does not greatly change the upper 50‐m dCo from the CTL model (Figure 6b compared with Figure 2a). Dust Co is assumed to have a solubility of 8% (Shelley et al., 2012), so the muted influence of dust Co is mostly due to the low mineral fraction of Co in dust (17.3 μg/g; Rudnick & Gao, 2014). These model results are consistent with observations and calculations showing small to nondetectable surface dust deposition effects in the Atlantic Ocean (Noble et al., 2012, 2017; Saito & Moffett, 2002; Shelley et al., 2012, 2016).

In contrast to dust, sediments are the major external driver of dCo distributions at the surface and in the ocean interior, with absolute dCo concentrations modified by over 50 pM (Figures 6c and 6d). Indeed, in this experiment surface Co drops to very low levels <10 pM when sediment Co supply is eliminated (NOSED; Figure 6e). In our model, there are two major components to sediment supply; one is the base sediment Co flux, and the second is the enhancement of Co fluxes at low oxygen levels. When the enhanced Co fluxes at low oxygen are removed (NOSEDOX), we can highlight regions where Co supply from sediments is mostly driven by this particular process (Figures 6f and 6g) and the ensuing influence on surface dCo (Figure 6h). By then comparing the results of NOSED with NOSEDOX, we can calculate the percentage influence of low oxygen sediment fluxes on dCo. This calculation shows that in the tropical ocean, over half of the influence of sediment Co supply is governed by low oxygen enhancing fluxes, whereas in the Arctic ocean, the strong sensitivity to sediment Co fluxes is driven by the large shelf areas (Figures 6i and 6j).

Our model does not include hydrothermal input of dCo. While this could be included in the model in a similar manner as for Fe (Tagliabue & Resing, 2016), there is no evidence for large basin‐scale dCo plumes alongside notable hydrothermal Fe signals (Hawco et al., 2016; Noble et al., 2012), despite observations of near‐field localized sources (Noble et al., 2017, and references therein).

### 3.3 Role of Internal Cycling

We also conducted a set of sensitivity experiments examining the role of various removal processes affecting dCo in the ocean interior. In all these experiments, the Co loss due to a specific process was removed, allowing us to examine how a given process contributes to maintaining the modeled dCo levels.

**Figure 7:**
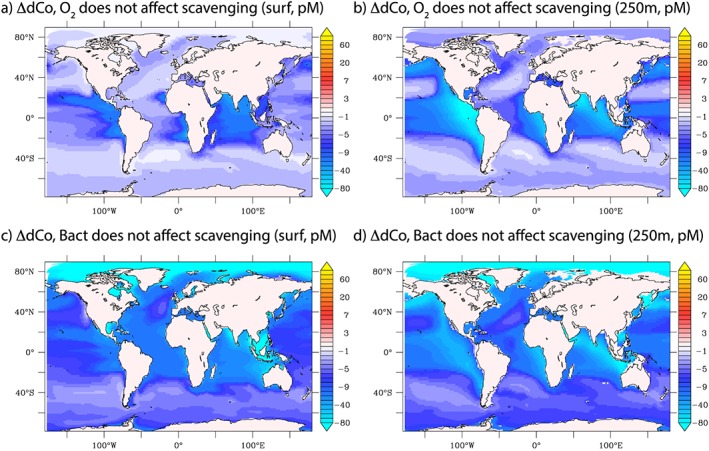
Absolute change in dCo (pM) at the surface and 250 m, when Co scavenging is not reduced at low oxygen levels (a and b) and not reduced by low rates of bacterial activity (c and d).

To first consider oxygen, Figures 7a and 7b show the impact of eliminating the reduction in scavenging by low O_2_ levels (equation (8)) at the surface and 250 m. In this experiment, scavenging rates are higher, and a clear effect in the tropical ocean emerges where low subsurface O_2_ levels contribute upward of 50 pM to the dCo signal (Figure 7b). In regions where low O_2_ zones in the ocean interior are coupled to the surface by upwelling, decreased Co scavenging at low O_2_ increases surface ocean dCo by up to 10–20 pM (Figure 7a). In the oxygen‐rich high‐latitude oceans, there is little effect of low O_2_ on lessening Co scavenging.

Eliminating the reduction in Co scavenging due to low levels of Mn‐oxidizing bacterial activity (equation (9) has a different pattern to O_2_ (Figures 7c and 7d). This parameterization for temperature‐based controls on scavenging was based on laboratory experiments with Mn‐oxidizing bacteria (Lee & Fisher, 1993) and field observations for limited dCo scavenging in the Ross Sea and under the sea‐ice (Noble et al., 2013; Saito et al., 2010). The Arctic Ocean now emerges as the strongest signal, both at depth and at the surface (dCo declines by more than 80 pM when variations in bacterial rates are ignored). When linked to the strong role for sediment Co supply in this region (section 3.2), this indicates that the low rates of bacterial activity in these cold waters permit sedimentary Co to have a greater influence on dCo levels. The low latitude ocean is also impacted by the greater rates of Co scavenging when low levels of bacterial activity are eliminated. The impact of bacteria is broadly similar to O_2_, but much more widespread, both at the surface and at depth.

### 3.4 The Southern Equatorial Pacific Co Plume: A Case Study

**Figure 8:**
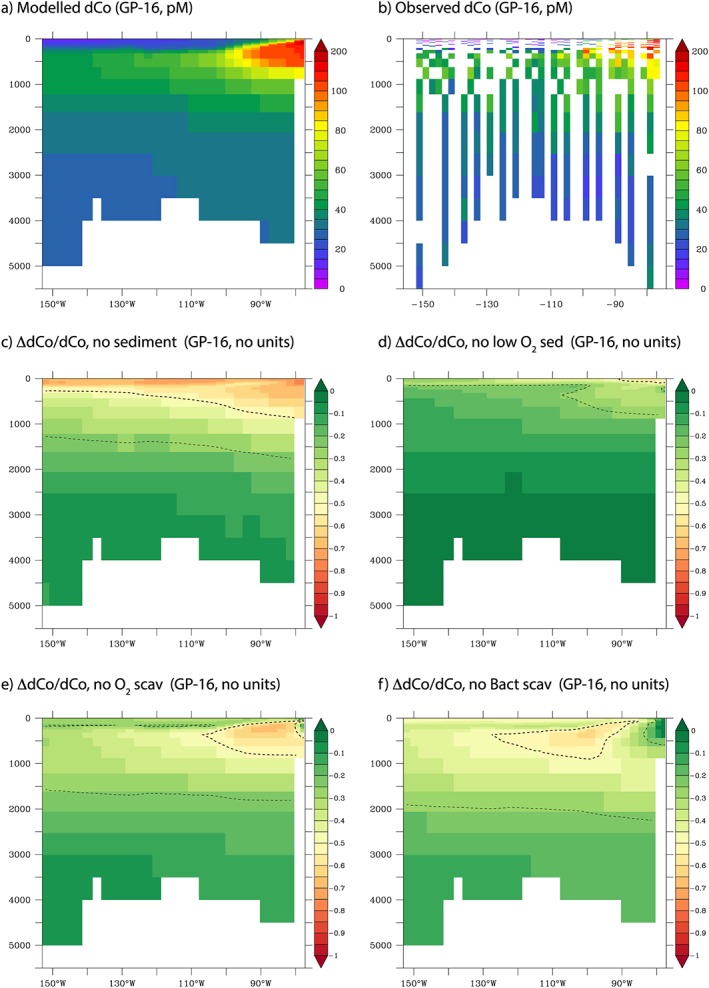
How different processes shape the Co plume observed on GP‐16. Top row shows (a) model and (b) data dCo (pM), while middle and bottom rows show the proportional change in dCo when there is (c) no sediment supply, (d) no enhanced sediment flux at low O_2_, (e) no reduction in scavenging at low O_2_, and (f) no reduction in scavenging at low rates of bacterial activity. Thick and thin contours highlight where a given process affects 50% and 25% of the magnitude of the dCo plume, respectively.

The GEOTRACES GP16 cruise to the southern equatorial Pacific observed a notable offshore dCo plume in the subsurface ocean emanating from the Peru margin (Hawco et al., 2016). Our new Co model provides a way in which to assess how external input and internal cycling processes govern this high Co feature. As seen previously, the model is able to reproduce the intensity and magnitude of the observed plume (Figures 8a and 8b) better than the low oxygen associated Atlantic plumes (see above). We use our suite of sensitivity tests to quantify by how much the dCo plume declines when sedimentary Co supply and the decreased scavenging of Co driven by low O_2_ and low rates of bacterial activity are removed. More than 70% of the dCo signal is eliminated by removing sedimentary Co supply very close to the margin, with the impact lessening further offshore (Figure 8c). The low O_2_ enhancement of sedimentary Co fluxes supports ~25% of the dCo plume (Figure 8d). The impact of low rates of scavenging due to the low O_2_ levels is muted very close to the margin but becomes much more important offshore (Figure 8e). This pattern is more marked for the role of low bacterial activity enhancing dCo levels, with little impact close to the margin but a greater impact offshore (Figure 8e). Thus, our model suggests that this dCo plume is initially controlled by high rates of sedimentary Co input close to the margin but that Co is then maintained in the dissolved pool by low rates of scavenging, first due to low O_2_ and then due to low rates of bacterial activity in the ocean interior.

## 4 A Synthesis of the Ocean Cobalt Cycle

**Figure 9:**
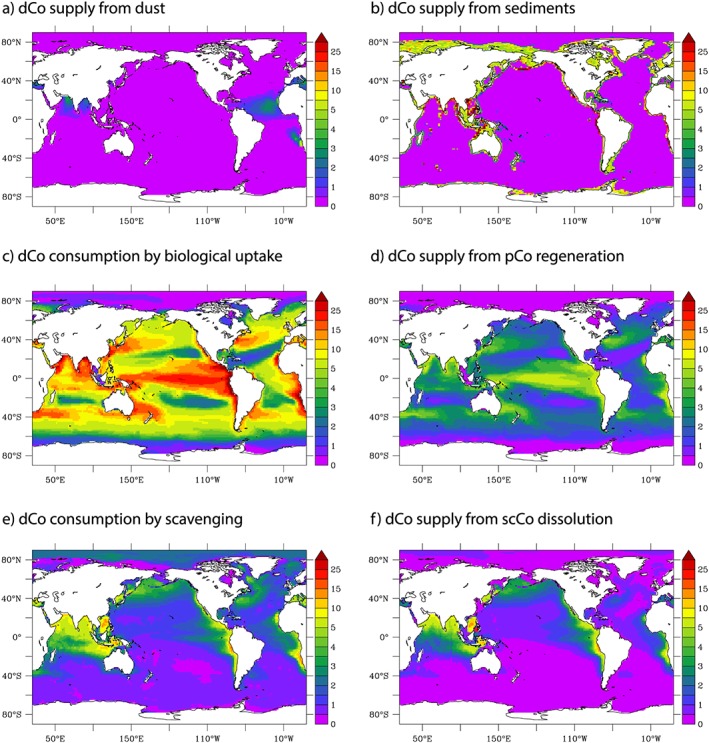
Total fluxes of dCo linked to various external sources (a and b) and internal cycling (c–f). All fluxes are depth integrated and are presented in units of μmol Co/m^2^ year_._

We can use our model to bring together the first synthesis of the major external inputs and internal cycling of Co in the global ocean. Figure 9 shows the vertically integrated fluxes of Co due to dust and sediment supply (Figures 9a and 9b), biogeochemical processes of phytoplankton uptake and regeneration (Figures 9c and 9d), and the scavenging and dissolution of scavenged Co (Figures 9e and 9f). What becomes apparent is the strong influence of sediment fluxes at ocean boundaries that must then be transported widely by low interior scavenging rates. In our model, almost two thirds of the total global sedimentary boundary flux of Co is driven by our parameterization of enhanced supply when low bottom water oxygen is low. This points to a need for further studies on how bottom water oxygen levels modulate Co sediment supply. Co loss due to phytoplankton uptake and resupply due to regeneration are unsurprisingly associated with typical patterns of ocean biological productivity (Figures 9c and 9d). In a similar manner to the spatial coupling between Co consumption by biology and regeneration, Co scavenging and dissolution are spatially linked (Figures 9e and 9f). It is notable that low O_2_ regions dissolve scavenged Co to dCo because of enhanced rates of scCo dissolution. This is not apparent in the higher O_2_ regions of the high latitudes. In these regions, for example, the Arctic, our model predicts that low temperatures decrease the activity of Mn‐oxidizing bacteria yielding a lower scavenging removal of dCo.

**Figure 10:**
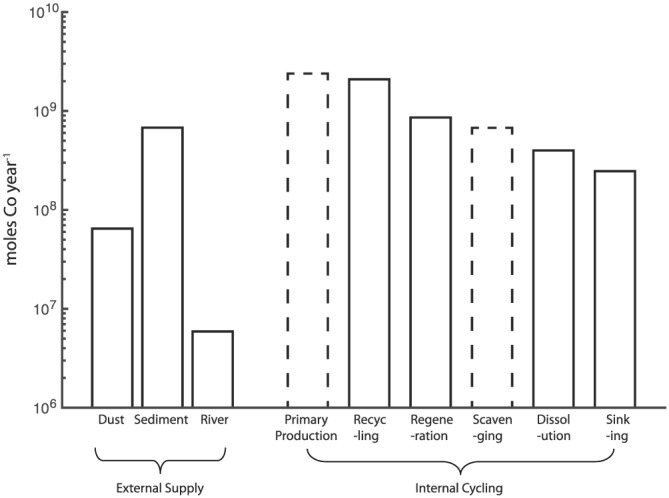
The magnitude of different processes in the modeled global cobalt budget (mol Co/year).

Our model is able to provide the first estimates of the major global fluxes shaping the oceanic cycle of Co and the ocean residence time of Co (Figure 10 and Table 3). Dust, sediments, and rivers supply 6.5 × 10^7^, 6.8 × 10^8^, and 5.7 × 10^6^ mol of Co annually. The sediment source compares favorably to an independent estimate (~6 × 10^8^ mol of Co annually) based on simpler calculations from field data sets (Hawco et al., 2017). Primary production consumes 23.9 × 10^8^ mol of Co, with much of this dCo sink balanced by recycling of 20.9 × 10^8^ mol from zooplankton, while regeneration of particulate organic Co resupplies a further 8.6 × 10^8^ mol each year. Globally, 3.1 × 10^8^ and 2.5 × 10^8^ mol of particulate Co (including organic and scavenged Co particles) sink across the 100‐ and 250‐m depth horizons each year, respectively. Scavenging removes 6.8 × 10^8^ mol from the dCo pool, and dissolution of scavenged Co returns 4.0 × 10^8^ mol/year. When combined with the total Co inventory of the ocean in our model (5 × 10^10^ mol), the total Co inputs of 7.5 × 10^8^ mol/year result in a global ocean Co residence time of 70 years. If this is split into upper 250 m and deeper than 250 m, then the residence times (ignoring physical exchanges) are approximately 7 years in the surface ocean and around 250 years deeper than 250 m (Table 3), similar to simpler early estimates (Bewers & Yeats, 1977; Saito & Moffett, 2002).

**Table 3:** Major Fluxes of the Modeled Cobalt Cycle

External inputs	Internal cycling	Value
Dust		6.5 × 10^7^ moles/year
Sediment		6.8 × 10^8^ moles/year
River		5.7 × 10^6^ moles/year
Total		7.5 × 10^8^ moles/year
	Primary production	24 × 10^8^ moles/year
	Recycling	21 × 10^8^ moles/year
	Regeneration	8.6 × 10^8^ moles/year
	Scavenging	6.8 × 10^8^ moles/year
	Dissolution	4.0 × 10^8^ moles/year
	Sinking PCo (250 m)	2.5 × 10^8^ moles/year
Global dCo inventory		5 × 10^10^ moles
Residence time (global)		250 years
Residence time (0–250 m)		7 years

The internal cycling of Co at the global scale is driven by different processes between the surface ocean and the ocean interior. Unsurprisingly, biological uptake and Co turnover by zooplankton are the major Co sink and source terms in the upper 250 m (23.9 × 10^8^ and 20.7 × 10^8^ mol/year) where they dominate over the scavenging sink (3 × 10^8^ mol/year). In addition to zooplankton recycling, resupply of dCo in the upper 250 m by particulate organic Co remineralization (8.5 × 10^8^ mol/year) is around double that from the dissolution of scavenged Co (4 × 10^8^ mol/year). Turning next to the ocean interior (>250 m), we find that dissolution of scavenged Co driven by low oxygen is the greatest dCo source globally (3 × 10^8^ mol/year) and is more than four times greater than remineralization of particulate organic Co (0.7 × 10^8^ mol/year, with zooplankton recycling reduced to 0.2 × 10^8^ mol/year). This switch in the dominant internal sources with depth is notable and may be unique to cobalt's biogeochemistry, with remineralization and recycling dominating in the upper water column and the dissolution of scavenged Co within the OMZs in the mesopelagic. Our emphasis on biological uptake in the upper 250 m agrees with a previous Co budget from the Atlantic Ocean (Dulaquais et al., 2014). However, in contrast to Dulaquais et al. (2014), we find dissolution of scavenged Co (putatively associated with Mn oxides) to be more important than organic Co remineralization in the ocean interior (deeper than 250 m) in our model. This difference likely reflects the fact that the work of Dulaquais et al. (2014) occurred in the relatively oxic Atlantic Ocean and dissolution of scCo will be much more important when the low oxygen zones of the Pacific and Indian Oceans are included (as in our global assessment). Our view is also consistent with observations of large dCo plumes within each of these major oxygen minimum zones (Hawco et al., 2016; Noble et al., 2012, 2017). Equally, it should be noted that particulate organic Co fluxes attenuate exponentially with depth, accounting for their greater importance in the upper 250 m and lesser role (in absolute terms) deeper than 250 m (Table 3). Finally, we highlight that these represent gross integrated fluxes from the model and a given Co atom may participate in more than one process during its lifetime in the ocean, for example, be remineralized from PCo, then scavenged to scCo and then dissolved back to dCo from scCo.

**Figure 11:**
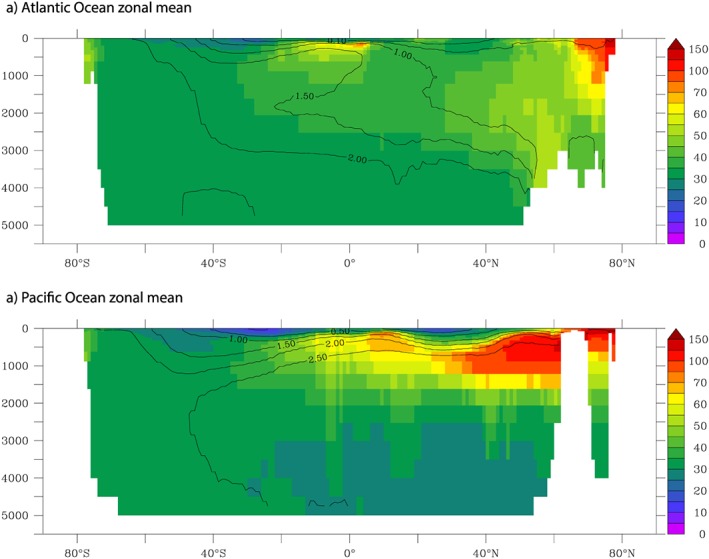
Zonal mean dissolved cobalt (pM) from the Atlantic and Pacific Oceans. Zonal mean PO_4_ (μM) is overlain as a contour.

Our model has provided us with a conceptual view of how Co is transported from boundary sources into the ocean interior. The southern equatorial Pacific case study suggested that a strong source must be coupled with low scavenging rates to facilitate transfer throughout the ocean. The model experiments show that direct Co supply by dust is negligible apart from some very local regions in the tropical Atlantic Ocean. In the equatorial latitudes of the Pacific and also the Atlantic Ocean, low O_2_ plays a key role in promoting Co transport by decreasing scavenging. This is seen by the imprint of high Co upon the meridional structure of the Atlantic and Pacific phosphate (PO_4_) distributions at low latitudes (Figure 11). Additional decoupling between Co and PO_4_ is observed at high latitudes in the Atlantic and Pacific. In the North Pacific, the model proposes an accumulation of dCo due to declining O_2_ in the oldest waters at intermediate water depth (which has some support in CLIVAR data, Figure 3f, and other North Pacific data sets; M. Saito personal communication, 2017), while in the North Atlantic, high Co from the Arctic is transported equatorward (Figure 11). The Arctic is O_2_ rich, compared to the low latitudes, and this region acts as a Co hotspot because high rates of Co input from the shallow shelves are coupled with low rates of removal due to cold temperatures depressing bacterial activity. In the Southern Ocean, shelves are narrower than in the Arctic, leading to lower Co input and little impact on dCo levels due to the scavenging loss in this highly oxic region. Ultimately, our model suggests that scavenging‐dissolution processes and their modulation by oxygen levels and bacterial activity are the key determinants of the oceanic distribution of Co. Future studies characterizing the chemistry and biology of Co scavenging are warranted, in particular, the generation of in situ estimates of kinetic scavenging rates, to better constrain this process.

## 5 Toward Quantifying the Biological Role of Cobalt

**Figure 12:**
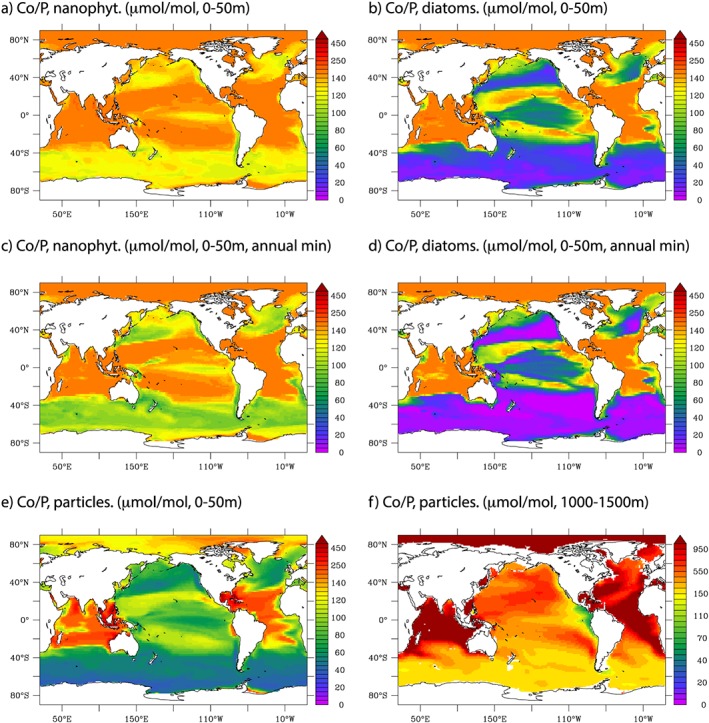
Annual mean Co/P quotas in nanophytoplankton and diatoms at 0–50 m (a and b). Annual minimum Co/P quotas in nanophytoplankton and diatoms at 0–50 m using monthly model output (c and d). Annual mean Co/P ratios in all particles for 0–50 and 1,000–1,500 m (e and f). All are in units of μmol Co/mol P.

In general, modeled phytoplankton Co quotas are lowest in the productive regions of the ocean and are highest in the oligotrophic gyres (Figures 12a and 12b). This reflects the fact that Co uptake in our model is independent of carbon and phosphorus (P) uptake, and thus, Co uptake can continue when growth rates (and C and P assimilation rates) are low. Due to the influence of Zn on Co uptake in diatoms, Co/P ratios are lowest for diatoms in the Zn‐rich Southern Ocean. Over the seasonal cycle, nanophytoplankton and diatom Co/P quotas can reach the minimum values of ~60 and <10 μmol/mol (Figures 12c and 12d) due to seasonal dCo depletion. Consistent with their overall low levels of phytoplankton biomass, absolute quantities of Co present in phytoplankton biomass are minimum in the oligotrophic gyres.

Modeled Co/P phytoplankton quotas reflect the observations compiled thus far from synchrotron X‐ray fluorescence methods (Twining et al., 2011, 2015; Twining & Baines, 2013). These data sets find Co/P quotas in the temperate Pacific Ocean of <50 μmol/mol for diatoms and >150 μmol/mol for nondiatoms (King et al., 2012), while in the Equatorial Pacific Ocean, Co/P quotas are <100 μmol/mol for diatoms and >150 μmol/mol for nondiatoms (Twining et al., 2011), and the CTL model is able to reproduce these limited observations (Figures 12a–12d). The model finds that the subtropical North Atlantic Ocean displays elevated Co/P quotas for both diatoms and nanophytoplankton, and these are broadly reflected in the elevated cell quotas of 50–170 μmol/mol measured along GA‐03 (Twining et al., 2015).

The Co/P ratio of the bulk particulate pool reflects the combination of the amalgamation of distinct assemblage quotas and any additional production of particulate Co via scavenging but does not include lithogenic Co. In general, the pattern (Figure 12e) represents that discussed previously for the phytoplankton. Co/P ratios are low (<100 μmol/mol) in regions of high growth rate and in the Southern Ocean where the dominant diatom demand for Co is repressed by elevated Zn levels. In contrast, Co/P ratios are greatest (>150 μmol/mol) in the tropical Atlantic and Indian Oceans. Notably this is without including any Co substitution within alkaline phosphatase of the dominant cyanobacteria populations in the model, which is suggested by observations of this metalloenzyme within regions of “accelerating” dCo:PO_4_ stoichiometries (Saito et al., 2016). Finally, it is noteworthy that the Co/P ratios increase strongly with depth due to the production of additional particulate Co from the interior ocean scavenging of dCo by Mn‐oxidizing bacteria (Figure 12f).

Ultimately, it is important to link the oceanic distributions and phytoplankton Co quotas to biological activity. At present, Co is known to have two major biological roles. First, vitamin B_12_ or cobalamin contains Co and is mainly required for the synthesis of the amino acid methionine and the nucleotide biosynthesis through the enzymes methionine synthase and ribonucleotide reductase, respectively (Bertrand et al., 2013; Rodionov et al., 2003). Second, it is known that Co can act as a substitute cofactor for Zn in carbonic anhydrase (Morel et al., 1994).

**Figure 13:**
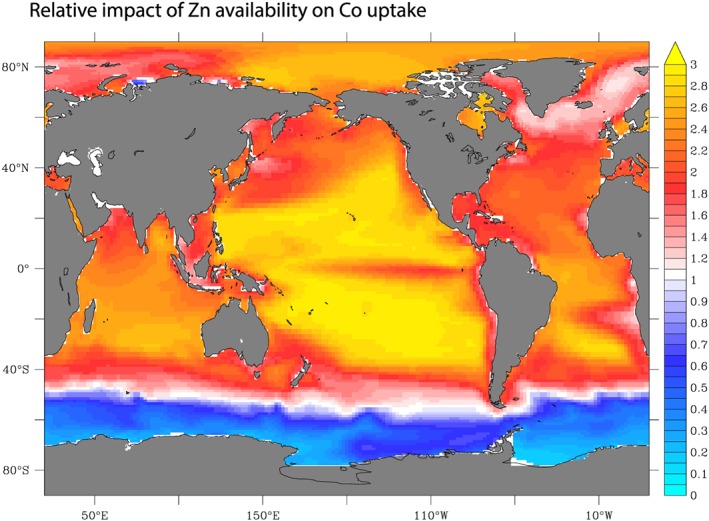
A map of the degree to which cobalt uptake is enhanced by Zn availability as per equation (6) (unitless).

In our model, we accounted for the impact of Zn on Co requirements via equation (6). The precise degree of upregulation or downregulation of phytoplankton Co uptake is largely unknown due to variations in the diversity of Zn/Co cambialism; hence, its parameterization is relatively subjective at this stage. Nevertheless, the direction of change across the surface ocean is driven by Zn availability and should be relatively robust. Figure 13 displays the relative change in Co uptake due to Zn (equation (6)) and shows that maximum impact of Zn on Co requirements should be occurring in the oligotrophic gyres of the Pacific Ocean as Zn is depleted, followed by the southern subtropical Atlantic and the northern subtropical Atlantic gyres. Moreover, Zn‐Co interactions may be further exacerbated in oligotrophic systems due to the connection between P scarcity and Zn/Co requirements that could explain high Co:P quotas in the surface Atlantic Ocean (Saito et al., 2016; Shaked et al., 2006). On the other hand, high levels of Zn in the Southern Ocean should lessen Co demands. Of course, this relies on the fact that we can broadly reconstruct Zn distributions from the close link between Zn and Si. In the future, it would be important to also develop a prognostic ocean Zn model that can be coupled to the current model.

At present, models such as PISCES do not account for vitamin regulation of phytoplankton physiology nor impact of Co or Zn scarcity affecting cellular enzymes. Instead, such global models tend to rely on identifying the most limiting resource that then governs carbon fixation rates. While some models are moving away from using the external nutrient concentration of resources to drive growth rates (Arteaga et al., 2014; Aumont et al., 2015), they still rely on a limited suite of resources and on “law of the minimum” parameterizations. In the future, it is important for models to expand their scope beyond N, P, Si, and Fe to consider other important resources, such as Co, that are known to be depleted in seawater (Moore et al., 2013) and to revisit the resource limitation parameterizations to account for the potentially important colimitation between different resources. By way of an example, we found that diatom Co dropped markedly in the NOSED and NOSEDOX experiments, highlighting how remote sources from ocean boundaries supports Co nutrition and also implying that changes in boundary sources and their propagation into the ocean interior due to past or future climate change may affect Co limitation.

## 6 Conclusions

Overall, our model does a good job in reproducing the growing data set of dCo measurements arising from the GEOTRACES and CLIVAR efforts and allows for some of the first global‐scale estimates of Co fluxes. We find an upper ocean residence time for Co of 7 years and a deep ocean residence time of 250 years, similar to previous estimates based on smaller data sets (Bewers & Yeats, 1977; Saito & Moffett, 2002). Our model highlights the sediments as the major external input of Co to the ocean and the importance of reduced scavenging removal in low oxygen regions such as the eastern tropical Pacific and cold regions such as the Arctic, in propagating Co throughout the ocean. The Arctic and Indian Oceans and low latitude upwelling systems are found to be the most Co‐rich regions of the ocean, with the Southern Ocean and then the oligotrophic gyres as the most Co poor. Therefore, these Co‐poor regions may be areas where Co has an impact on biological activity. Representing the impact of Co on microbial vital rates will, however, require a greater level of detail in the modeling of phytoplankton physiology in global models to account for resource substitution and colimitation. Such advances will shed important insights on metal quotas in marine phytoplankton given any future changes to external inputs and internal cycling of micronutrients.
